# Tracking-Based Denoising: A Trilateral Filter-Based Denoiser for Real-World Surveillance Video in Extreme Low-Light Conditions †

**DOI:** 10.3390/s25175567

**Published:** 2025-09-06

**Authors:** He Jiang, Peilin Wu, Zhou Zheng, Hao Gu, Fudi Yi, Wen Cui, Chen Lv

**Affiliations:** 1 School of Information and Control Engineering, China University of Mining and Technology, Xuzhou 221116, China; jianghe@cumt.edu.cn (H.J.); zhengzhou@cumt.edu.cn (Z.Z.); guhao@cumt.edu.cn (H.G.); yifudi@cumt.edu.cn (F.Y.); cuiwen@cumt.edu.cn (W.C.); 2 School of Software, Taiyuan University of Technology, Taiyuan 030024, China

**Keywords:** video denoising, trilateral filter, amplitude-phase filter, low light, surveillance video

## Abstract

Video denoising in extremely low-light surveillance scenarios is a challenging task in computer vision, as it suffers from harsh noise and insufficient signal to reconstruct fine details. The denoising algorithm for these scenarios encounters challenges such as the lack of *ground truth*, and the noise distribution in the real world is far more complex than in a normal scene. Consequently, recent state-of-the-art (SOTA) methods like VRT and Turtle for video denoising perform poorly in this low-light environment. Additionally, some methods rely on raw video data, which is difficult to obtain from surveillance systems. In this paper, a denoising method is proposed based on the trilateral filter, which aims to denoise real-world low-light surveillance videos. Our trilateral filter is a weighted filter, allocating reasonable weights to different inputs to produce an appropriate output. Our idea is inspired by an experimental finding: noise on stationary objects can be easily suppressed by averaging adjacent frames. This led us to believe that if we can track moving objects accurately and filter along their trajectories, the noise may be effectively removed. Our proposed method involves four main steps. First, coarse motion vectors are obtained by bilateral search. Second, an amplitude-phase filter is used to judge and correct erroneous vectors. Third, these vectors are refined by a full search in a small area for greater accuracy. Finally, the trilateral filter is applied along the trajectory to denoise the noisy frame. Extensive experiments have demonstrated that our method achieves superior performance in terms of visual effects and quantitative tests.

## 1. Introduction

Reducing the noise inherent in video sensors is a critical challenge, particularly under the harsh conditions of low-light surveillance. The demand for reliable, high-quality video from surveillance systems is ever-increasing, being driven by security needs such as preventing nighttime theft. This quality is also directly essential for applications like autonomous driving, object detection, and action recognition. This necessitates effective denoising techniques that can operate under severe signal degradation. The principle of video denoising is to reconstruct the true signal corrupted by this sensor noise. This is achieved by exploiting the spatiotemporal redundancy inherent in the video data stream. The method involves identifying patches with high similarity to a target region across both space and time. A weighted combination of these similar patches is then used to reconstruct the original feature, effectively restoring the clean signal from its noisy frames. The challenges originate directly at the sensor level. First, the scarcity of incident photons in low-light environments results in a fundamentally low signal-to-noise ratio [1,2,3,4] and significant signal distortion. Second, unlike the sophisticated sensors in professional cameras from manufacturers like Sony or Panasonic, the sensors in cost-effective surveillance hardware are inherently more prone to thermal and read noise, thus severely amplifying issues when the captured signal itself is weak. ﻿

A low-light video denoising method relies on raw video data as input [5]. However, these raw data are difficult to obtain from standard surveillance systems, which typically output processed and compressed formats like H.264 or YUV/RGB. Attempting to reverse the on-camera Image Signal Processing (ISP) pipeline to recover the raw data often leads to severe artifacts and information loss, as critical sensor-level information is irrevocably discarded during processing. A recent method [6] divides surveillance video data into moving and static areas, aiming to separate static and moving regions. It introduces an optimization solution based on Dynamic Mode Decomposition (DMD) and Plug-and-Play Alternating Direction Method of Multipliers (PnP-ADMM), which involves a minimization problem equation and incorporates an implicit regularization term to reduce noise. However, it fails to effectively remove noise in extremely low-light and high-noise scenarios. Moreover, it primarily focuses on dynamic mode decomposition, neglecting structure and texture. Additionally, DMD relies on linear system approximations, which can be inadequate for complex, nonlinear motion, and high-frequency DMD modes may be over-smoothed, adversely affecting details. Another video denoising method [3] embeds the BM3D [7] algorithm into the HEVC processing workflow, which reduces redundant computations by replacing BM3D [7]’s block matching with HEVC’s motion estimation. This approach is considered efficient for video denoising but also faces challenges in extremely noisy low-light conditions, as such hybrid frameworks often prioritize computational efficiency over adaptive noise modeling, leading to insufficient robustness against complex noise patterns in practical surveillance scenarios.

**Figure 1:**
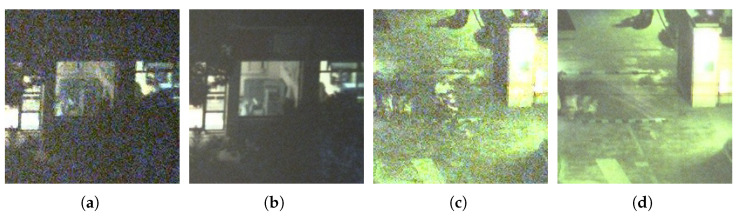
Comparison of noisy images in video and their corresponding temporal average. (**a**) A single noisy frame from the static sequence. (**b**) The result of averaging adjacent frames in the static region. (**c**) A single noisy frame from the moving sequence. (**d**) The result of averaging adjacent frames in the moving region.

In this paper, we address the task of denoising surveillance videos from low-light environments. This task has the following difficulties. First, we lack *ground truth*. Second, the noise distribution in each RGB channel is different. Third, the noise varies greatly across frames, and the low-light environment further complicates the problem. Fourth, the images obtained by the many well-known denoising algorithms are either too smooth or a little noisy, which indicates that it is difficult to provide a good balance between noise removal and detail retention in such a harsh environment. In the experiment, noise is largely removed after averaging the adjacent frames. In Figure 1, the first two images contain only stationary objects, whose noise is largely reduced by multi-frame temporal averaging. The second two images include moving objects, but the moving electronic bike (as shown in Figure 1d) disappears and leaves a trajectory blur. Inspired by this phenomenon, we believe that if the trajectory of the moving object is accurately found, and we filter along the trajectory, the noise will be well suppressed, and reversal artifacts can be efficiently reduced like [8]. In this paper, a tracking-based denoising algorithm is proposed, and our contributions can be summarized in the following aspects:

1. A simple but efficient motion vector estimation method is put forward, which can be applied to another computer vision task.
2. A motion updating filter called an amplitude-phase filter is proposed, which improves the accuracy of these motion vectors. In addition, a denoising filter, namely the trilateral filter, is proposed by considering the gradient information, and it can suppress the gradient reversal artifact of the bilateral filter.
3. A tracking-based video denoising method is proposed.

## 2. Related Work

### 2.1. Traditional Methods

Early methods, like content-adaptive filtering [9], aimed to suppress noise variance in flat areas while maintaining crisp edge delineation through minimizing the weighted least square error. Subsequently, wavelet-based algorithms [10,11] demonstrated superior capabilities in distinguishing signal and noise components, while they tend to introduce artifacts. While the Kalman filter-based method [12] primarily targets the reduction of temporal flicker artifacts, its reliance on linear system assumptions fundamentally limits its effectiveness in real-world low-light scenarios characterized by nonlinear noise, resulting in inconsistent denoising performance.

Later, researchers also proposed patch-based techniques to exploit temporal coherence. For instance, ref. [13] uses optical flow for motion-compensated patch matching for denoising. Other representative methods like VBM3D [14] and its extension VBM4D [15] utilize block matching and collaborative filtering in higher-dimensional transform domains for noise removal. However, VBM4D’s [15] primary bottleneck is its computationally intensive motion estimation, which is required for grouping spatiotemporal volumes. This process lacks the efficiency of VBM3D’s [14] staged transform approach, leading to a significant increase in complexity. The method in [16] models groups of similar spatiotemporal patches within an empirical Bayesian framework, avoiding motion estimation errors by not relying on motion compensation. This approach leverages the low intrinsic dimensionality of patch groups to enhance denoising but depends heavily on noise characteristic assumptions and may face challenges in accurately estimating statistics due to the high dimensionality of spatiotemporal patches and limited sample sizes. Even some more recent deep learning approaches that also leverage patch-based strategies such as those proposed in [17,18,19] can still suffer from long running times despite their different underlying mechanisms.

A method specialized for low-light surveillance video denoising in [20] employs a combination of filtering techniques and adaptively estimates noise levels. By conducting denoising recursively using only the previous frame, it is well suited for hardware implementation due to its efficiency. Its primary limitation, however, arises from its core reliance on a Kalman filter for temporal prediction. This filter inherently assumes a linear motion model, which is often violated in dynamic surveillance scenarios involving abrupt or complex movements. Consequently, this can lead to motion blur and ghosting artifacts around fast-moving objects, undermining the algorithm’s performance in complex scenes. Additionally, the method is formulated for grayscale video, limiting its direct application to color surveillance systems.

The method in [21] employs a multi-stage pipeline including a Kalman filter and nonlocal means (NLM). Although its low memory footprint makes it suitable for resource-constrained hardware, the architecture suffers from a major drawback: its sequential processing and computationally intensive NLM stage create an inherent performance bottleneck, resulting in high latency. This intrinsic slowness, combined with the need for extensive hyperparameter tuning and its reliance on a linear motion assumption, limits its effectiveness in complex, real-time scenarios.

### 2.2. Supervised Deep Learning Methods

The rise of Convolutional Neural Networks (CNNs) [22] has dramatically enhanced video denoising performance. Several studies [23,24,25,26,27,28] have explored their potential. For example, ref. [23] consists of two stages: spatial denoising followed by a motion compensation stage to reduce flickering. While effective, the reliance on optical flow in such methods [23,27,29,30] comes at a steep computational price. Unlike coarser block-matching techniques that estimate a single displacement for a patch, optical flow computes a dense motion vector for every pixel. This per-pixel granularity is computationally intensive, rendering these methods too slow and costly for practical deployment. Furthermore, their accuracy is compromised under challenging conditions like low light and high noise, where the foundational assumption of brightness constancy is often violated. To enhance performance, various architectures have been proposed, including U-Net-inspired designs [24,26] and multi-scale feature extraction frameworks [31]. A prominent example, FastdvdNet [24], employs a U-Net architecture with depthwise separable convolutions to implicitly handle motion, thereby avoiding the costly estimation of optical flow. However, its architecture relies solely on standard convolutional layers, lacking mechanisms like attention or advanced feature fusion. This structural simplicity limits its ability to capture long-range temporal dependencies, making it less effective for scenes with significant motion.

Recursive Neural Network (RNN)-based algorithms [32,33,34,35] exhibit excellent performance in utilizing temporal modeling and leveraging inter-frame correlations. FloRNN [32], for example, is designed for online processing by replacing the true backward pass of a bidirectional RNN with a finite ’look-ahead’ module. While this design enables real-time operation, it introduces a fundamental asymmetry: context from past frames is propagated directly via recurrence, while future context is an approximation derived from a limited look-ahead window and then warped back to the current frame. This indirect estimation of future information is inherently less robust than a true bidirectional pass. The approach in [33] uses stacked RNN layers to capture temporal dynamics, and EMVD [35] introduces multi-stage fusion to reduce computational load, though this may limit their capacity to model long-range dependencies. The sequential nature of RNNs creates a core architectural dilemma: a choice between maximum accuracy with slow, offline bidirectional models and speed with faster but less robust online approximations. This trade-off fundamentally limits their practical application in real-time systems.

In contrast, transformer-based methods [4,27,36,37] achieve superior denoising accuracy but come with high computational demands. Video Restoration Transformer (VRT) [27] exemplifies such trade-offs. While its Temporal Mutual Self-Attention mechanism achieves state-of-the-art performance, the model relies on window-based attention for implicit motion modeling, which limits its ability to capture long-range temporal dependencies. This constraint becomes particularly evident in the presence of large motions, where the model struggles to efficiently model distant frame relationships and fully leverage motion information, potentially compromising restoration quality. To mitigate these issues, VRT processes videos in segmented clips and employs sequence shifting—strategies that add architectural complexity and computational overhead. Similarly, the work in [4], which focuses on low-light environments, integrates 3D Shifted Window Transformer blocks within a U-Net-like encoder–decoder architecture. This design cleverly avoids explicit motion estimation modules like optical flow. However, its primary limitation lies in its final temporal fusion stage. This module relies on a simplified, implicit alignment, using a single similarity score to re-weight entire feature maps before fusion via convolution. While computationally simpler than dedicated alignment techniques, this soft re-weighting approach may be insufficient for handling large or complex motions, potentially leading to motion blur or ghosting artifacts. This architecture, combined with the deep stack of 3D attention blocks, still results in significant computational and memory overhead.

### 2.3. Unsupervised Methods

In the realm of unsupervised video denoising [25,38,39,40], recent deep learning approaches have explored diverse architectures to address spatiotemporal noise without clean data supervision. Recent approaches, such as UDVD [25], employ “blind-spot” convolutions [41] to model intricate spatiotemporal relationships. By using rotated and asymmetric kernels, this method enhances feature representation. However, its performance degrades in scenarios involving large-scale, rapid motion. This limitation stems from a constrained search area, which is confined to orthogonal axes (e.g., below and right) while overlooking diagonal orientations like the upper left. Another approach, Temporal As a Plugin (TAP) [39], extends pre-trained image denoisers by inserting trainable temporal modules into their skip connections. These modules use deformable convolutions for motion alignment, simplifying the transition from image to video denoising. However, a significant domain gap arises when applying a model trained on standard, well-lit images to complex low-light video. The image denoiser backbone, optimized for simple noise patterns, struggles to handle severe degradations such as signal-dependent noise and motion blur commonly found in low-light conditions. Crucially, its inaccurate denoising results introduce residual noise and artifacts into the generated "pseudo-clean" frames, which misguide the learning of temporal modules and lead to error propagation and amplification. Turtle [42] also introduces a novel framework based on U-Net, but it employs a truncated causal history mechanism. It uses Cross-Frame Attention to implicitly align features and Cross-Channel Attention to aggregate features from historical frames. However, its core design choice to use only a truncated history of past frames imposes a significant limitation. By design, the model has no access to future frames, creating an information bottleneck. This hard truncation restricts the model’s receptive field in the temporal dimension, preventing it from capturing long-range dependencies.

Building on unsupervised learning, self-supervised methods like [43,44,45] further minimize reliance on paired data. While [25] explores untrained denoising networks, self-supervised video methods directly address temporal noise by leveraging noisy video itself for training. The approach in [45] utilizes the translation equivariance of Fully Convolutional Networks (FCNs) and a progressive fine-tuning strategy. By learning from “pseudo-clean” data, it can theoretically achieve a lower noise level than methods that learn directly from noisy data. However, unlike in normal lighting scenarios, the noise type and intensity in low-light conditions are harsh and not easy to estimate. This indirectly affects the performance of both the pre-trained model and the final output.

In summary, recent SOTA methods, including TAP [39], Turtle [42], and VRT [27], all demonstrate limited robustness in low-light scenarios. Although their performance is commendable in daylight, it degrades substantially under low-illumination conditions with severe noise, where they struggle with effective noise removal. Furthermore, these deep learning methods inherently suffer from the common disadvantage of high memory consumption.

## 3. Methods

**Figure 2:**
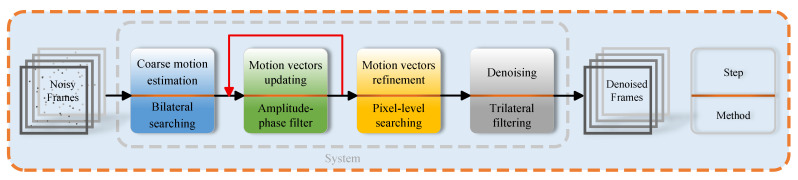
Flowchart of our method. The process begins with coarse motion estimation to generate initial motion vectors, which are then iteratively updated and refined. Finally, a trilateral filter produces the clean frames.

This work is a significantly extended version of our conference paper [46]. In this section, we will introduce our tracking-based algorithm shown in Figure 2.

### 3.1. Coarse Motion Estimation

**Figure 3:**
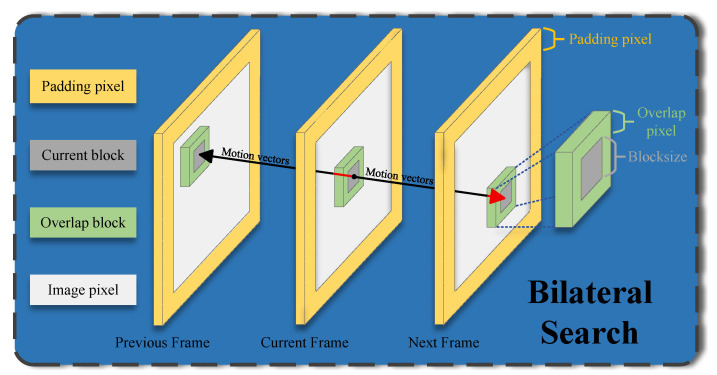
Coarse motion estimation via bilateral search. The search areas in the previous and subsequent frames are positioned symmetrically with respect to the location of the current block.

The coarse motion estimation module is founded upon the classic Block-Matching Estimation (BME) framework, as exemplified by the method in [47]. This approach is widely adopted for its computational efficiency. Our primary contribution in this context is not the invention of the search mechanism itself, but its novel application and adaptation to the domain of low-light surveillance video denoising, which is a departure from its conventional use in Frame Rate Up-Conversion (FRUC). The objective is also fundamentally different: while in FRUC, motion vectors are primarily used for interpolating missing frames via methods like Overlapped Blocks Motion Compensation (OBMC), our implementation leverages them to enhance the robustness of cross-frame matching specifically for denoising purposes. We selected this bilateral search strategy because it is not only computationally efficient due to the simple Sum of Absolute Differences (SAD) matching criterion, but it also provides accurate results. It can effectively avoid artifacts such as overlaps or holes that can result from unidirectional motion estimation. Furthermore, the resulting motion vectors are well suited for the subsequent vector refinement stage. The process of our bilateral search is illustrated in Figure 3.

In this process, each frame is segmented into 8×8 blocks, where 8 is the block size (Bs). To improve matching robustness, we adopt (OBMC), which extends the boundary of the original block by a margin of Overlapped pixels ($O_{p}$). To facilitate the processing of these edge blocks, the frame is padded with a certain width of Padding pixels (Pp). Based on these steps, the search area is defined as $A_{ab}=\left\{\left(x,y\right)|1+\left(a-1\right)\times Bs+Pp-Op\leq x<a\times Bs+Pp+Op,1+\left(b-1\right)\times Bs+Pp-Op\leq y<b\times Bs+Pp+Op\right\}$, where subscripts *a* and *b* denote the block’s row and column indices, respectively. Naturally, the ranges of these indices are constrained by the dimensions of the input video frame (the resolution of motion vectors field), which we denote as M×N. The lower bound for both *a* and *b* indices is 1, and the upper bound is determined by the frame’s boundaries. Specifically, for index *a*, it must satisfy $1\leq a\times B_{s}+P_{p}+O_{p}<M$. Once the block’s spatial range is defined, SAD is used to calculate the pixel differences.

(1) $$SAD=\sum_{x,y\in A_{ab}}\sum_{\Delta x,\Delta y\in SA_{ab}}\left|F_{n-1}\left(x-\Delta x,y-\Delta y\right)-F_{n}\left(x+\Delta x,y+\Delta y\right)\right|$$

In Equation (1), $F_{n-1}\left(x-\Delta x,y-\Delta y\right)$ represents a block with the starting point (x−Δx,y−Δy) in the previous frame, and $F_{n}\left(x+\Delta x,y+\Delta y\right)$ is defined analogously for the subsequent frame. $SA_{ab}$ is the search area in the previous frame, and $SA_{ab}=\left\{\left(\Delta x,\Delta y\right)|-Sws\leq \Delta x<Sws,-Sws\leq \Delta y<Sws\right\}$, in which Δx and Δy are integer offsets, and Sws denotes the Search window size. The motion vector (Δx,Δy) that minimizes the SAD value is selected, as shown in Equation (2).

(2) (Δx,Δy)=argminSAD

### 3.2. Motion Vector Updating

The initial motion vector (Δx,Δy) is often coarse, and thus an update mechanism is introduced. We represent (Δx,Δy) in terms of its amplitude and phase, where amplitude $A=\sqrt{\Delta x^{2}+\Delta y^{2}}$ and phase $P=\arctan(\frac{\Delta y}{\Delta x})$. Therefore, the vector can be modified by altering *A* and *P*, transforming it as follows: $\left(\Delta x,\Delta y\right)=\left(A\cos P,A\sin P\right)\to \left(A^{\prime }\cos P^{\prime },A^{\prime }\sin P^{\prime }\right)=\left(\Delta x^{\prime },\Delta y^{\prime }\right)$.

**Figure 4:**
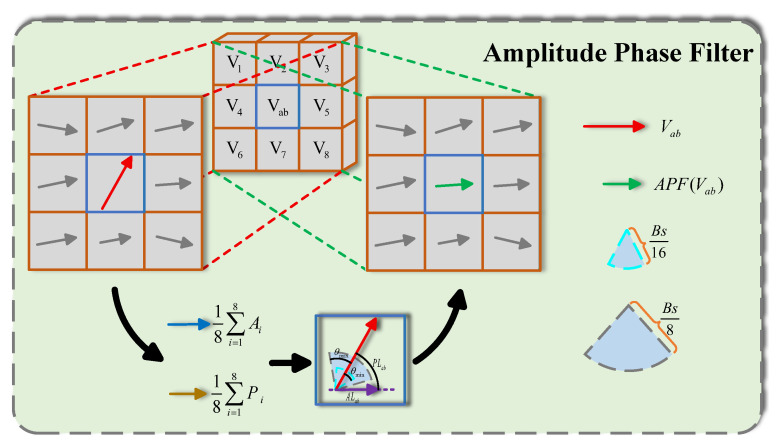
The Amplitude-Phase Filtering process. The filter first computes the difference between each motion vector and the average of its eight neighbors. This difference is then thresholded to validate the vector’s reasonableness. Finally, an iterative median filter is applied to ensure spatial smoothness.

The mechanism, therefore, is designed to transform (A,P) to $\left(A^{\prime },P^{\prime }\right)$. Let $V_{ab}=\left(\Delta x,\Delta y\right)$ be the vector to be updated, which belongs to the block in the *a*th row and the *b*th column, and $V_{1}-V_{8}$ are its 8-neighbor vectors, as illustrated in Figure 4.

To reduce singular motions, the local continuity of the motion vector field is considered. Local continuity is measured as the Manhattan distance in terms of amplitude ($AL_{ab}$) and phase ($PL_{ab}$), as defined in Equation (3). Here, $A_{ab}$ and $P_{ab}$ are the amplitude and phase of vector $V_{ab}$. Similarly, $A_{i}$ and $P_{i}$ are the amplitude and phase of the neighbor vector $V_{i}$, respectively, where *i* ranges from 1 to 8.

(3) $$AL_{ab}=|A_{ab}-\frac{1}{8}\sum_{i=1}^{8}A_{i}|\;PL_{ab}=\left|P_{ab}-\frac{1}{8}\sum_{i=1}^{8}P_{i}\right|$$

A Rationality matrix *R* is used to judge whether the current motion vector is reliable. Since it is a binary classification problem, the element $R_{ab}$ in this matrix can only be 0 or 1, and $R_{ab}$ indicates the rationality of the block at the *a*th row and the *b*th column. Equation (3) is used to estimate the motion vector $V_{ab}$ as well as calculating the local continuity in amplitude $AL_{ab}$ and phase $PL_{ab}$. Finally, Equation (4) is used for the rationality matrix initialization. In Equation (4), $\theta_{max}$ and $\theta_{min}$ are the maximum and minimum phase offset thresholds.

(4) $$R_{ab}=\left\{\begin{array}{cc} 0 & \;\;AL_{ab}\geq \frac{Bs}{8},\;PL_{ab}\geq \theta_{max}\\ 1 & \;\;AL_{ab}\leq \frac{Bs}{16},\;PL_{ab}\leq \theta_{min} \end{array}\right.$$

Vectors with a rationality of 1 are considered reasonable, while those with a rationality of 0 are deemed unreasonable. For reasonable vectors, we leave them unchanged. But for unreasonable vectors, a median filter is applied to update them. This process is called the Amplitude-Phase Filter (APF), and Equation (5) shows the details.

(5) $$APF\left(V_{ab}\right)=\left\{\begin{array}{cc} median\{V_{1},V_{2},V_{3},\ldots ,V_{8}\} & \;R_{ab}=0\\ V_{ab} & \;R_{ab}=1 \end{array}\right.$$

Furthermore, a stop condition of this iterative mechanism must be designed. Our design follows two principles: the number of updates cannot be excessively large, and the majority of motion vectors must be reasonable. The first principle can be easily satisfied by setting the maximum number of iterations. The second condition can be measured by the sum of the rationality matrix, namely $\sum_{a=1}^{M}\sum_{b=1}^{N}R_{ab}$. In Equation (6), M×N is the resolution of the motion vector field as mentioned before, and β ranges between 0 and 1, which describes the percentage of reasonable vectors in all vectors.

(6) $$\sum_{a=1}^{M}\sum_{b=1}^{N}R_{ab}\geq \beta MN$$

Our proposed APF introduces a novel approach to motion vector updating. Unlike the approach in [48], which relies on the Bidirectional Prediction Difference and subsequent Outlier Detection, our classification is performed directly using the amplitude and phase of the motion vectors. This allows for a more nuanced evaluation of vector reliability, as the phase component explicitly accounts for directional variations in motion. Moreover, our adaptive, iterative update offers a more flexible solution than the fixed two-stage (median-then-mean) smoothing process in [48] without introducing significant computational overhead.

### 3.3. Motion Vector Refinement

**Figure 5:**
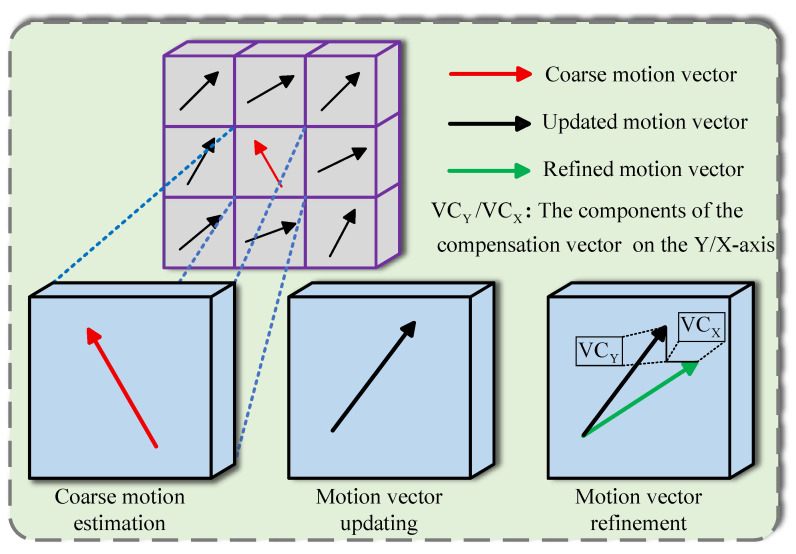
Motion vector refinement. A final adjustment is performed on the vectors using an exhaustive search within a small, local window.

After the second step, the motion vectors may not be precise enough, and their accuracy cannot reach the pixel level without a full search. A full search is often avoided by many methods due to its time complexity. However, its idea can be adopted. Our approach is to compensate for the motion vectors by doing full search only in a small area. In this way, we can obtain more accurate motion vectors, and the small area helps the system avoid significant computational cost, as shown in Figure 5.

Suppose a vector (Δx,Δy) is initialized by coarse motion estimation, and it is then updated to $\left(\Delta x^{\prime },\Delta y^{\prime }\right)$. After the final refinement step, the vector becomes $\left(\Delta x^{\prime }+VC_{X},\Delta y^{\prime }+VC_{Y}\right)$, as $\left(VC_{X},VC_{Y}\right)$ is the compensation vector on the X/Y-axis obtained from a full search in a small area.

(7) $$\begin{array}{cc} \; & SAD_{p}=\sum_{x,y\in A_{ab}}\sum_{VC_{X},VC_{Y}\in SSA_{ab}}|F_{n-1}(x-\Delta x^{\prime }-VC_{X},\\ & y-\Delta y^{\prime }-VC_{Y})-F_{n}\left(x+\Delta x^{\prime }+VC_{X},y+\Delta y^{\prime }+VC_{Y}\right)| \end{array}$$

In Equation (7), $SAD_{p}$ is short for the Sum of the Absolute Difference, and the footnote *p* denotes pixel-level precision. $F_{n-1}\left(x-\Delta x^{\prime }-VC_{X},y-\Delta y^{\prime }-VC_{Y}\right)$ represents an image block $F_{n-1}$ starting at $\left(x-\Delta x^{\prime }-VC_{X},y-\Delta y^{\prime }-VC_{Y}\right)$ in the previous frame, and $F_{n}\left(x+\Delta x^{\prime }+VC_{X},y+\Delta y^{\prime }+VC_{Y}\right)$ is defined analogously.

$SSA_{ab}$ is a new search area, which is different from the search area $SA_{ab}$. First, $SA_{ab}$ is the search area used in vector initialization, and its size should be large to improve the likelihood of capturing a wide range of motions. However, $SSA_{ab}$ is the small search area used in vector compensation. Second, the search method used in $SA_{ab}$ is bilateral search, but the search method in $SSA_{ab}$ is a full search. In fact, $SSA_{ab}=\left\{\left(VC_{X},VC_{Y}\right)|-SSws\leq VC_{X}<SSws,-SSws\leq VC_{Y}<SSws\right\}$, where SSws here denotes small search window size.

The final optimal vector $V_{ab}^{*}$ can be acquired using Equation (8), and $V_{ab}^{*}$ is the best vector for the block in row *a* and column *b*. The entire process for motion vector estimation, updating, and refinement is summarized in Algorithm 1.

(8) $$V_{ab}^{*}=\left(\Delta x^{\prime }+VC_{X},\Delta y^{\prime }+VC_{Y}\right)=\arg\min SAD_{p}$$

**Algorithm 1** Motion vectors estimation, updating, and refinement.**Require:** Low-light Surveillance Video $I\in R^{m\times n\times t}$, Block Size Bs=8, Padding Pixel Pp=16, Overlap Pixel Op=4, Search Window Size Sws=6, Vector Updating Number num=0, Rationality Matrix R, Reasonable Degree β=0.96, and Small Search Window Size SSws=2.**Ensure:** The Final Vector V and Rationality Matrix R.
1:  Initialization: $M=\frac{m}{Bs}$, $N=\frac{n}{Bs}$, Zero Matrix $V\in R^{M\times N\times (t-1)}$, and $R\in R^{M\times N\times (t-1)}$2:  **for** each k∈[2,t] **do**3:      **for** each a∈[1,M] **do**4:          **for** each b∈[1,N] **do**5:             Get the areas $A_{ab}$, $SA_{ab}$, and $SSA_{ab}$.6:             Solve Equation (2) to get the vector (Δx,Δy).7:             **Repeat:** Compute $AL_{ab}$, $PL_{ab}$ using Equation (3).8:             Update $R_{ab}$, $V_{ab}$ using Equations (4) and (5).9:             num=num+1, and $R\left(a,b,k-1\right)=R_{ab}$.10:            **While:** $\frac{\sum_{a=1}^{M}\sum_{b=1}^{N}R_{ab}}{MN}\geq \beta$ or num=15.11:           Get the updated vector $\left(\Delta x^{\prime },\Delta y^{\prime }\right)$.12:           Solve Equation (8) to get the best vector.13:           $V\left(a,b,k-1\right)=\left(\Delta x^{\prime }+VC_{X},\Delta y^{\prime }+VC_{Y}\right)$.14:           Update $R_{ab}$ using Equation (5) for each vector.15:           $R\left(a,b,k-1\right)=R_{ab}$.16:        **end for**17:    **end for**18:**end for**19:**return** The final vector **V** and rationality matrix **R**;

### 3.4. Trilateral Filter

**Figure 6:**
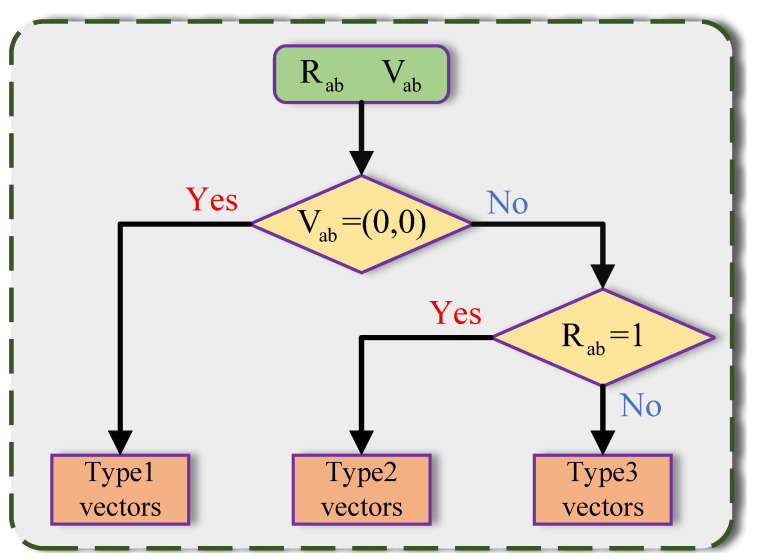
Motion vectors classification. Each motion vector is classified into one of three types based on $R_{ab}$ and $V_{ab}$.

Motion vectors can be classified into three categories using the criteria depicted in Figure 6, namely type 1, type 2 and type 3 vectors. Type 1 vectors are zero vectors; thus, only stationary objects can be found in their corresponding blocks. Type 2 vectors are not zero vectors but they are reasonable, meaning their corresponding blocks have found well-matched counterparts in the adjacent frames. Type 3 vectors are correspondingly unreasonable, signifying that their corresponding blocks fail to find the matching pairs in adjacent frames. Since the trilateral filter is based on the bilateral filter [49], we consider that a bilateral filter is an edge-preserving filter, and it can preserve the image’s edge features while denoising. However, the bilateral filter has two disadvantages. The first is its slow speed, which has already been solved by methods [50]. The second is that the images filtered by a bilateral filter [49] have the gradient reversal artifact.

To address this artifact, we propose an innovative trilateral filter designed to suppress the gradient reversal artifacts often associated with bilateral filters. While conventional bilateral filters operate on the spatial and pixel domain, and other trilateral filters like those in [51,52] have incorporated third domains such as depth edges or motion vector similarity, our method uniquely introduces a gradient similarity weight. This design choice is specifically tailored to our task of video denoising, as it effectively preserves texture and fine details that are critical in the restored images. Inspired by this concept, our trilateral filter considers three aspects of information: the spatial domain, the pixel domain, and the gradient domain.

The spatial domain information is measured by the local Euclidean distance in Equation (9)), and $w_{s}\left(a,b\right)$ is the similarity coefficient for the block in row *a* and column *b*. $\rho_{s}$ is a constant, which is related to the block variance $\delta_{s}^{2}$, and it equals $\frac{1}{2\delta_{s}^{2}}$. $\rho_{p}$ in Equation (10) and $\rho_{g}$ in Equation (11) have similar meanings.

The pixel domain information is measured by the Euclidean distance between the pixel values. In Equation (10), $w_{p}\left(a,b\right)$ is adopted as the similarity metric for the block in row *a* and column *b* in the pixel domain.

The gradient domain information is measured by the Euclidean distance between the gradient images, namely $gSAD_{p}$. In Equation (12), $w_{g}\left(a,b\right)$ is the gradient-domain similarity constant for the block in row *a* and column *b*, while $G_{n-1}$ and $G_{n}$ are the gradient of blocks $F_{n-1}$ and $F_{n}$. In the experiment, (1,−1) and ${\left(1,-1\right)}^{T}$ are used for the computation of gradients along the *x*-axis and *y*-axis.

(9) $$w_{s}\left(a,b\right)=\exp\left(-\rho_{s}\sum_{i=1:8}{\left(V_{ab}^{*}-V_{i}\right)}^{2}\right)$$

(10) $$w_{p}\left(a,b\right)=\exp\left(-\rho_{p}|SAD_{p}{|}^{2}\right)$$

(11) $$w_{g}\left(a,b\right)=\exp\left(-\rho_{g}{\left|gSAD_{p}\right|}^{2}\right)$$

(12) $$\begin{array}{cc} \; & gSAD_{p}=\sum_{x,y\in A_{ab}}\sum_{VC_{X},VC_{Y}\in SSA_{ab}}|G_{n-1}(x-\Delta x^{\prime }-VC_{X},\\ & y-\Delta y^{\prime }-VC_{Y})-G_{n}\left(x+\Delta x^{\prime }+VC_{X},y+\Delta y^{\prime }+VC_{Y}\right)| \end{array}$$

The final coefficient $w\left(a,b\right)=w_{s}\left(a,b\right)\times w_{p}\left(a,b\right)\times w_{g}\left(a,b\right)$. This coefficient integrates the information of the spatial domain, pixel domain, and gradient domains of an image, and it is therefore more robust. In addition, $\sum_{x,y}w\left(a,b\right)=1$ and $0<\sum_{x,y\in A_{ab}}\sum_{VC_{X},VC_{Y}\in SSA_{ab}}w\left(a,b\right)<1$; thus, w(a,b) is suitable as a weighting factor for the block in row *a* and column *b*. Based on these, trilateral filter TF(a,b) for vector $V_{ab}^{*}$ is defined in Equation (13), in which ∗ means a starting point $\left(x+\Delta x^{\prime }+VC_{X},y+\Delta y^{\prime }+VC_{Y}\right)$ in block $F_{n}$.

(13) $$TF\left(a,b\right)=\frac{\sum_{x,y\in A_{ab}}\sum_{VC_{X,Y}\in SSA_{ab}}F_{n}\left(*\right)w\left(a,b\right)}{\sum_{x,y\in A_{ab}}\sum_{VC_{X,Y}\in SSA_{ab}}w\left(a,b\right)}$$

The trilateral filter suppresses noise differently for each vector type shown in Figure 6. For type 1 vectors (zero vectors), noise is reduced by averaging 10 adjacent frames. Type 2 vectors are denoised directly using Equation (13). For type 3 vectors, nonlocal similarity and a large search window (BSws) are employed to find matching pairs, after which Equation (13) is applied for denoising.

## 4. Experiments

### 4.1. Datasets for Real-World Low-Light Surveillance Video Denoising

The evaluation of denoisers for low-light surveillance video heavily relies on representative datasets, yet existing benchmarks present significant limitations for this specific task. While widely used benchmarks such as Set8 [23] and DAVIS [53] exist, they primarily feature well-lit common scenarios and are thus unsuitable. Several datasets have been proposed specifically for low-light environments, including CRVD [54], and those in [4,5]. CRVD [54] provides 1080p videos from an IMX385 sensor across five ISO levels, while [5] targets even more extreme conditions (<0.1 lux) at 4K resolution. However, both datasets generate motion by manually controlling static objects frame by frame, resulting in discontinuous motion patterns confined to indoor environments, which fail to capture the fluidity of real-world dynamics. Although the dataset in [4] offers more realistic and complex motion models across its 210 video pairs, it shares a fundamental issue with CRVD [5,54]: they are all provided in RAW format. This creates a critical domain gap, as consumer-grade surveillance cameras (Hikvision, Dahua) typically output compressed streams like YUV or H.264 due to hardware and bandwidth constraints. Attempting to reverse-engineer RAW data from these formats via an inverse ISP pipeline is an ill-posed problem that introduces substantial estimation errors. Compounding these issues, the datasets from [4,5] are not publicly available. Finally, while the public DID dataset [55] offers multi-camera diversity and is accessible, it is designed for video enhancement, and its dynamics are generated by camera motion across static scenes, making it inappropriate for evaluating denoising on scenes with independent object motion.

To address this, we collected real-world extreme low-light sequences with resolution 1920 × 1072 using Hikvision DS-IPC surveillance cameras. The sequences feature wide-angle residential views with static and slow traffic and top–down road perspectives with fast vehicles, encompassing complicated motions and challenging backgrounds. Our dataset is provided in the RGB color space, matching the typical output of surveillance systems and thus eliminating the domain gap associated with RAW-based datasets, and it comprises 14 video clips (around 800 noisy samples) covering static and dynamic regions for quantitative evaluation, which is shown in Table 1 and Table 2. Equations (14)–(16) show the definitions of PSNR and SSIM. In Equation (14), MAX=255, and MSE is defined in Equation (15). In Equation (15), m×n is the resolution of the *ground truth* image $I_{gt}$. $I_{denoise}$ is the denoised result of the input noisy image. In Equation (16), *x* and *y* are two signals and $\mu_{x}$, $\mu_{y}$ are their average values. $\sigma_{x}$, $\sigma_{y}$ are variance *x* and *y*, and $\sigma_{xy}$ is the covariance of *x* and *y*. $c_{1}$, $c_{2}$ are small values, and both of them equal 0.001. They serve to stabilize the division when the denominators are close to zero.

(14) $$PSNR=10\times log_{10}\left(\frac{MAX^{2}}{MSE}\right)$$

(15) $$MSE=\frac{1}{mn}\sum_{i=1}^{m}\sum_{j=1}^{n}{\left(I_{gt}\left(i,j\right)-I_{denoise}\left(i,j\right)\right)}^{2}$$

(16) $$SSIM=\frac{\left(2\mu_{x}\mu_{y}+c_{1}\right)\left(2\sigma_{xy}+c_{2}\right)}{\left(\mu_{x}^{2}+\mu_{y}^{2}+c_{1}\right)\left(\sigma_{x}^{2}+\sigma_{y}^{2}+c_{2}\right)}$$

**Figure 7:**
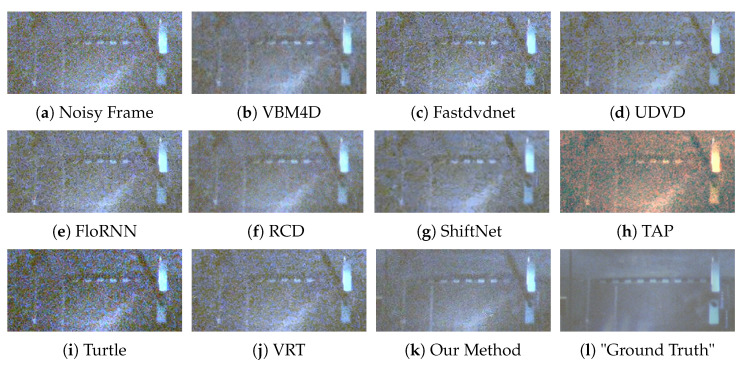
Qualitative comparison on a dynamic scene with moving barriers.

**Figure 8:**
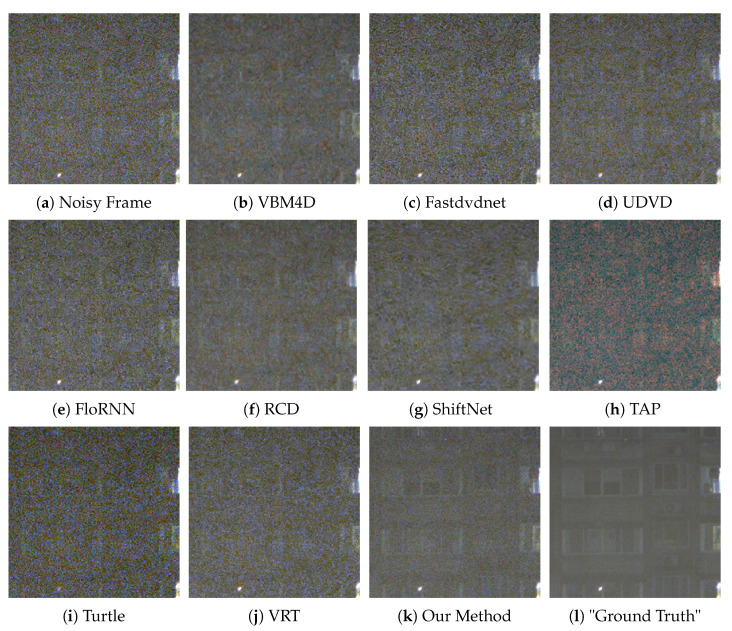
Qualitative comparison on a static scene with residential buildings.

**Figure 9:**
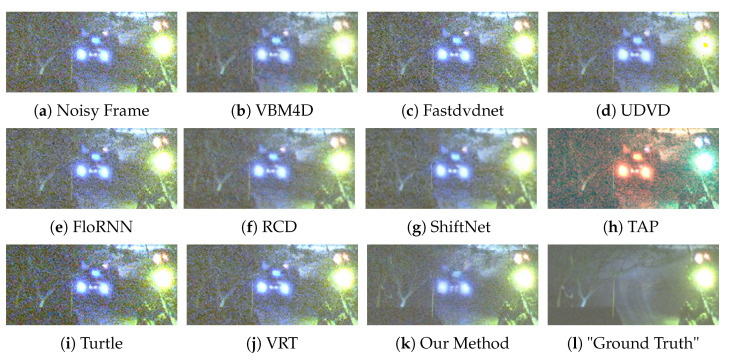
Qualitative comparison on a dynamic scene with moving car.

**Figure 10:**
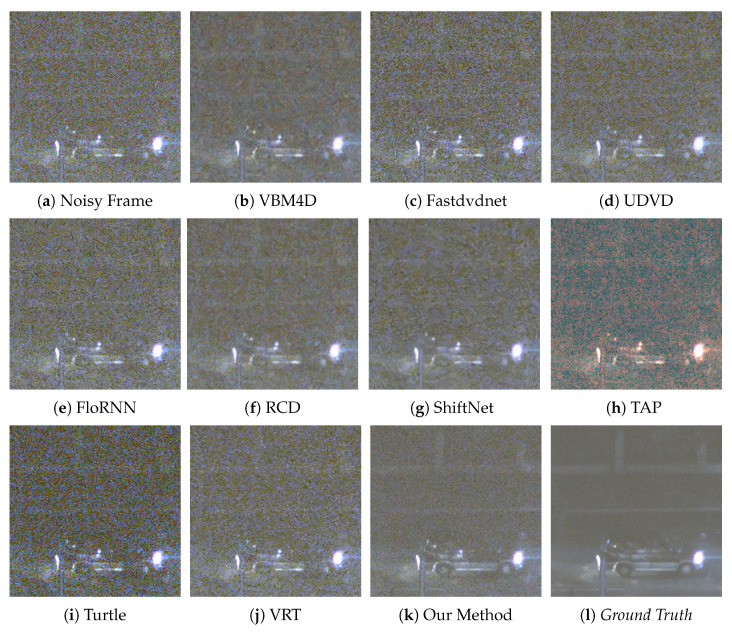
Qualitative comparison on a static scene with static vehicle.

**Figure 11:**
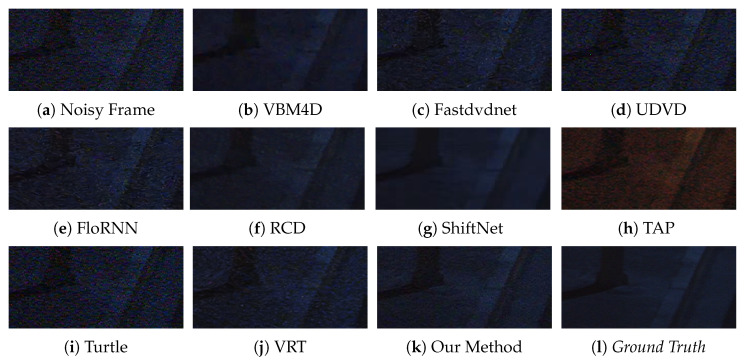
Qualitative comparison on a static scene with roadside staircase.

**Figure 12:**
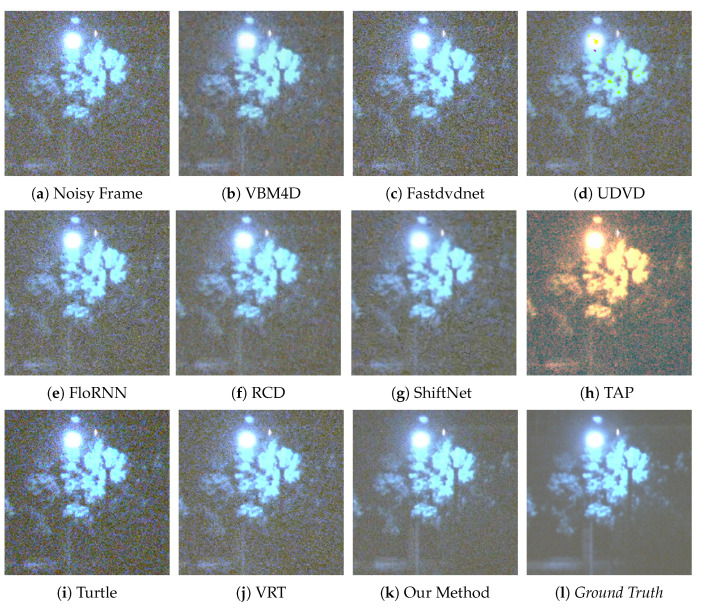
Qualitative comparison on a static scene with tree and street lamp.

We provide six visual results for qualitative comparison in Figure 7, Figure 8, Figure 9, Figure 10, Figure 11 and Figure 12. Static areas use pseudo-GT via multi-frame averaging; dynamic areas lack reliable GT due to motion blur invalidating temporal averaging. This provides a stringent testbed for evaluating denoising robustness on actual surveillance artifacts.

### 4.2. Implementation Details

To evaluate the denoising performance of our method, nine popular denoising methods are used to make comparisons with our method, namely VBM4D [15], FastdvdNet [24], UDVD [25], FloRNN [32], RCD [27], ShiftNet [28], TAP [40], Turtle [42], and VRT [27].

The experimental setup employed Matlab R2023a and PyCharm 2023.1 (python3.8, CUDA11.3) as the primary software environments. The hardware configuration included a single NVIDIA V100-SMX2-32GB GPU, a 12-core Intel Xeon Platinum 8255C CPU operating at 2.50 GHz, and 43 GB of system memory. All of the source code is available on Github, and we follow the default parameters. We use PSNR and SSIM as they are widely used evaluation metrics in the video denoising. PSNR mainly measures the pixel-wise error between two images, while SSIM mainly measures the structural similarity between two images in video.

### 4.3. Quantitative Tests and Visual Evaluations

Table 1 and Table 2 show the average values of PSNR and SSIM of 14 video sequences. In both PSNR and SSIM tests, our algorithm performs best in all video sequences, demonstrating that our method can retain the structural features of each frame effectively and it can suppress the pixel-wise error of each frame to some extent.

The final denoising performance also needs to be judged by the human eye; thus, the visual quality of each denoised image is important. A low-light environment is filled with the noise of different categories; therefore, in the process of noise removal, the high-frequency information, such as image edge and texture, can easily to be mistaken for the noise and then suppressed by the denoising algorithm. For instance, in Figure 8, our method sharply delineates the edges and contours of the window, whereas results from competing methods are still plagued by complex noise artifacts. In Figure 10, our approach adeptly restores the structure of the car, rendering its wheels and the overhead fence distinctly discernible. In contrast, TAP [39] exhibits severe color distortion, and other algorithms also yield suboptimal outcomes. VBM4D [15], for example, achieves noise reduction but at the expense of sacrificing fine details. Finally, in Figure 12, several methods show limitations. UDVD [26] introduces noticeable green artifacts on the tree, and TAP [39] once again suffers from severe color deviation. While Turtle [42] maintains structural details, it visually amplifies the noise.

Although ShiftNet [28] and RCD [27] achieve commendable results, our method demonstrates a superior trade-off, producing a perceptibly cleaner result that better preserves the tree’s intricate structure. In general, in the low-light environment, our method has a strong ability to protect image structures, and it is also robust to the environment with extremely low luminance.

### 4.4. Speed

Speed is an important metric for evaluating an algorithm. As shown in Table 3, our method is not the fastest compared to other algorithms, achieving approximately 1.17 s to process a single frame. These tests were all conducted on a system equipped with an RTX 4090D GPU with 24 GB VRAM, paired with an AMD EPYC 9754 128-Core Processor (18 vCPUs utilized, max frequency 3.1 GHz) and 60 GB of system memory, using Python 3.8, and CUDA 11.3. However, our proposed method operates on a CPU and does not leverage specialized hardware like GPUs, which is in contrast to many contemporary deep learning techniques that rely on GPUs for computational acceleration. It is noteworthy that the majority of the time consumption in our algorithm arises from search operations, which are relatively independent and thus amenable to GPU acceleration. We intend to investigate GPU-accelerated versions in future work.

### 4.5. Intensity Curve

**Figure 13:**
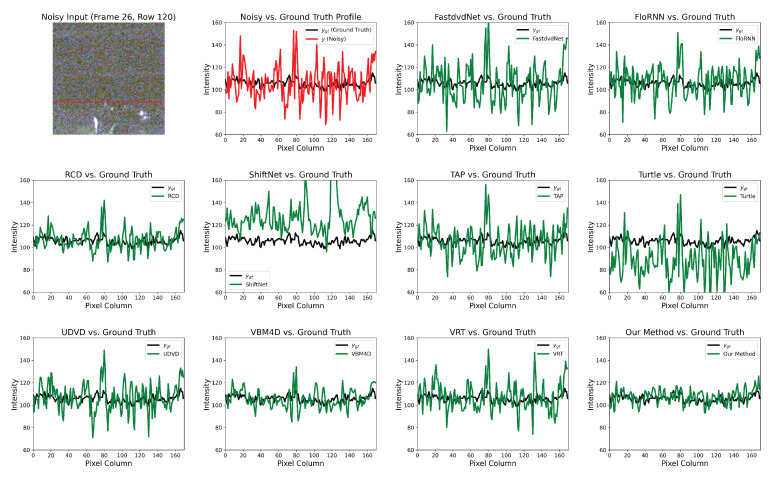
One example of the intensity curve. Our method demonstrates the most stable and effective noise removal, as evidenced by its signal (green line) being in the closest alignment with the ground truth (black line).

The intensity curve is a visualization method. Suppose $F\in R^{m\times n\times 3}$ is a frame in the noisy video, and $Y\in R^{m\times n}$ is its luminance channel. A single line of pixels $y\in R^{1\times n}$ is sampled from Y. Similarly, the same line from the denoised results and *ground truth* are also extracted. In Figure 13, the red curve y is a noisy signal from row 120 of frame 26 in video sequence 2, while the black signal $y_{gt}\in R^{1\times n}$ is the *ground truth*.

The green curve represents the denoising result for each method. It can be seen that deep learning-based methods, such as FastdvdNet [24], ShiftNet [28], and Turtle [42], perform poorly on low-light videos. This is because in such a harsh environment, it is difficult to accurately model the complex noise distribution solely through the adjustment of network weights, leading to insufficient smoothing. Moreover, the intensity curve of the traditional VBM4D [15] is overly smoothed, and significant residual noise remains around pixel column 80. This indicates a loss of detail, which is corroborated by the texture of the staircase in Figure 11. Finally, the overall difference between our curve and $y_{gt}$ is the smallest, and its profile follows the *ground truth* more closely and smoothly, which again proves that our algorithm achieves the best denoising performance.

### 4.6. Video Denoising Performance on DAVIS and CRVD Benchmarks

**Figure 14:**
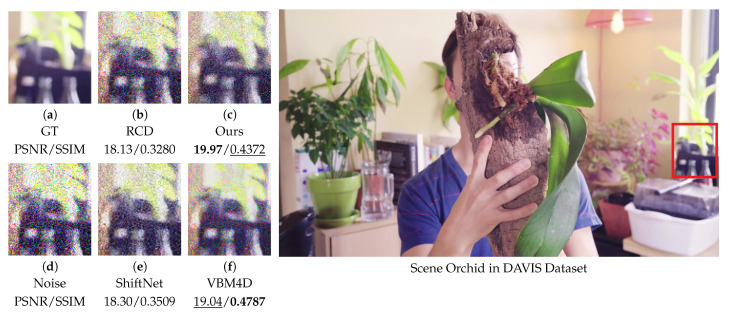
Comparison of the orchid scene from the DAVIS [53] dataset. Visual comparison of methods on a zoomed-in patch (left) with metrics; red box in the image (right) shows the patch location.

To further validate the generalizability and robustness of our algorithm, we also evaluated its performance on standard benchmark datasets. Additional experiments were carried out on the DAVIS [53] and CRVD [54] datasets. For CRVD [54], we converted the RAW data to RGB format to ensure a fair comparison. For DAVIS [53], we followed common practice in video denoising by adding Gaussian noise with a standard deviation of 50. We compared our method against several representative top-performing approaches from our main experiments—VBM4D [15], ShiftNet [28], and RCD [27]—using both qualitative and quantitative evaluations. Representative results are presented below under the same evaluation protocol as in our main study. As shown in Figure 14, which depicts flowers and their pots, our method performs competitively, effectively suppressing prominent color noise artifacts while achieving performance comparable to other state-of-the-art methods. In the results, **bold** and underlined values indicate the best and second-best performance, respectively.

**Figure 15:**
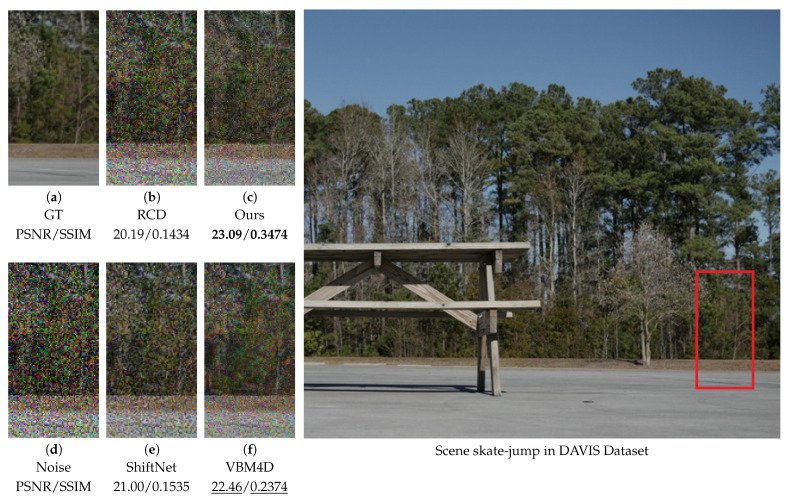
Comparison of the skate-jump scene from the DAVIS [53] dataset. Visual comparison of methods on a zoomed-in patch (left) with metrics; red box in the image (right) shows the patch location.

As shown in Figure 15, our algorithm achieves the highest PSNR and SSIM values, particularly in regions where the ground and foliage intersect. However, we observe that VBM4D [15] preserves fine textures slightly better, resulting in less blurring. This can be explained by the continuous camera motion in this scene, which results in an absence of static regions. Since our method employs distinct strategies for static and dynamic areas, the lack of static regions necessitates a motion-oriented approach throughout the entire frame, leading to a minor performance trade-off. The superior quantitative results of our approach are likely attributable to its enhanced capability to suppress dominant color noise, which significantly influences PSNR and SSIM metrics.

**Figure 16:**
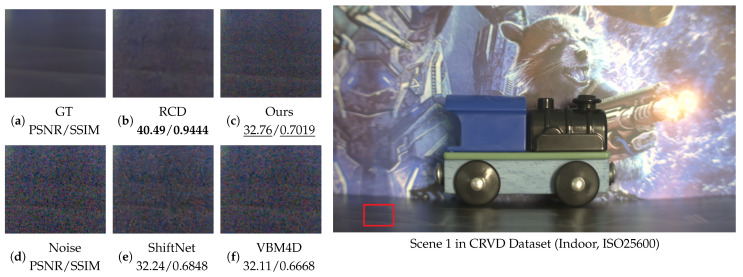
Comparison of Scene 1 from the CRVD [54] dataset. Visual comparison of methods on a zoomed-in patch (left) with metrics; red box in the image (right) shows the patch location.

Figure 16 presents a detailed view of the ground texture from Scene 1 of the CRVD dataset [54].

To ensure consistency across experiments, the RAW data from CRVD [54] were converted to the RGB color space using a standard linear transformation. This conversion follows well-established industrial imaging pipelines and may introduce minor color shifts, yet it does not significantly influence the denoising performance comparison. Since CRVD [54] includes both ground-truth and noisy image pairs, no synthetic noise was introduced. Although the dataset involves a static camera setup consistent with our application scenario, it does not represent a low-light or complex-noise environment. In this setting, our method achieves the second-best performance, effectively preserving horizontal ground textures. While the visual smoothness of our result is slightly inferior to that of RCD [27], our approach demonstrates significantly better noise suppression capability compared to both ShiftNet [28] and VBM4D [15].

**Figure 17:**
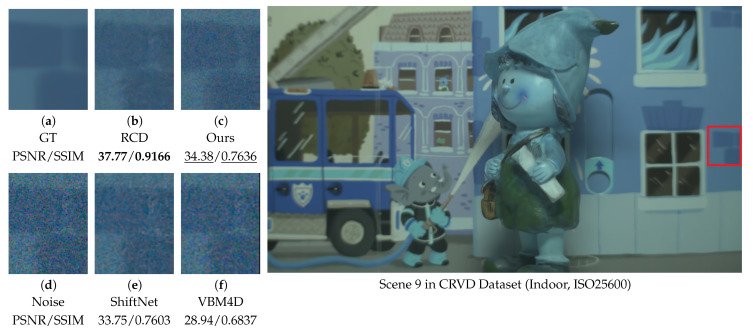
Comparison of Scene 9 from the CRVD [54] dataset. Visual comparison of methods on a zoomed-in patch (left) with metrics; red box in the image (right) shows the patch location.

Figure 17 displays the wall texture from Scene 9, in which our method again achieves the second-best performance. It successfully preserves the fine white gaps between tiles while providing effective noise removal. In summary, while our algorithm is specifically optimized for denoising low-light surveillance videos with complex real-world noise, its competitive performance on general benchmark datasets demonstrates that it is not narrowly specialized. These results indicate that our approach maintains strong generalization capability across different scenes and noise characteristics.

## 5. Model Analysis

### 5.1. Why Low-Light Environment Is Extremely Harsh?

**Figure 18:**
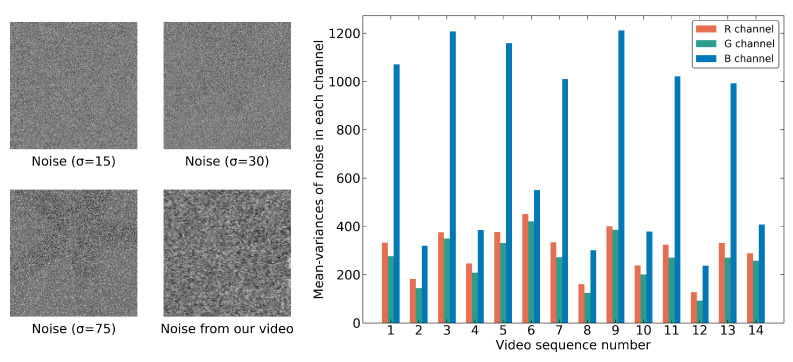
The average variance of RGB channels across video sequences and noisy grayscale images confirms the extremely harsh noise in our low-light surveillance environment.

**Figure 19:**
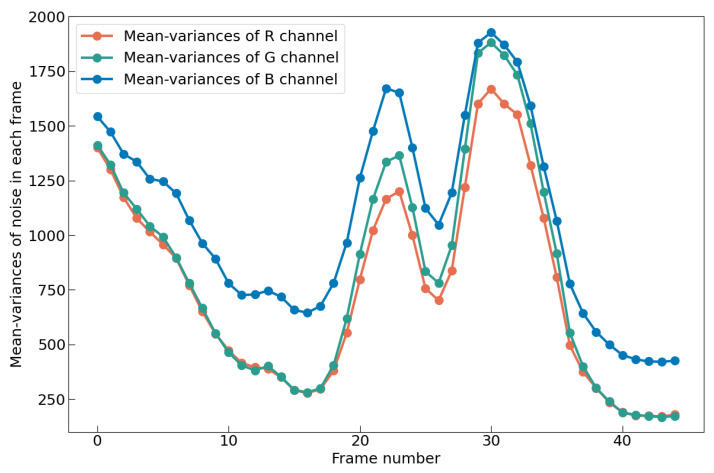
The average variances of RGB channels across frames in Video 2, demonstrating significant noise level fluctuations over time due to complex environmental conditions.

In the traditional denoising tasks, Gaussian noise is artificially added to an image. Usually, the variance is used to measure the noise complexity, with larger variances indicating more complex noise. The variance of Gaussian noise is set as 15, 25, 30, 50, 75, etc. Gaussian noise with a variance of 75 is considered to have a complex distribution. However, noise in the real world is far more complex than Gaussian noise, and a low-light environment intensifies this complexity. Our 14 video sequences are divided into different RGB channels, and then the mean-variance of noise in each channel is calculated, and Figure 18 and Figure 19 show the statistical results. In Figure 18, the mean variance of noise in each color channel is different, and all the mean variances are much larger than 75. In Figure 19, noise varies from frame to frame, and the variation is almost random. In other words, the noise in such an environment is constantly changing spatially and temporally, which undoubtedly increases the denoising difficulty.

### 5.2. Why Our Model Can Work?

**Lemma** **1.** *Matrix* **D*** is made up of K random values $d_{1}-d_{K}$, and * **D** *can be decomposed into the sum of L symmetric matrices without considering the zero values in * **D***, namely * **D** *= $D_{1}+D_{2}+\ldots D_{L}$ and L≤K.*

**Proof.** 

*Case 1:* If L=K, we set $D_{i}=\left[\overset{i-1}{\overset{︷}{0,\cdots ,0}},d_{i},\overset{K-i}{\overset{︷}{0,\cdots ,0}}\right]$, 1≤i≤K; thus, $D=D_{1}+D_{2}+\cdots +D_{K}$, and the proof ends.
*Case 2:* If L<K, a technique called mathematical induction is used, and this technique consists of three steps.

- Step 1: When number n=1, we set K=1, and $D=[d_{1}]$, which meets the lemma.
- Step 2: When number n=K, and if the lemma is satisfied, that is, $D=\left[d_{1},d_{2},\cdots ,d_{K}\right]=D_{1}+D_{2}+\cdots +D_{L}$, and L<K.
- Step 3: Based on step 2, and when number n=K+1, $D=[d_{1},d_{2},\cdots ,d_{K},d_{K+1}]$. We set $D_{L+1}=\left[\overset{K}{\overset{︷}{0,\cdots ,0}},d_{K+1}\right]\Rightarrow D=D_{1}+\cdots +D_{L}+D_{L+1}$ and L+1<K+1; thus, the lemma holds when n=K+1 if it holds when n=K. By the property of mathematical induction, this lemma holds for all natural numbers.

For instance, if $D=[d_{1},d_{2},d_{3},d_{4}]$, three symmetric matrices, namely $D_{1}=\left[d_{1},d_{2},d_{2},d_{1}\right]$, $D_{2}=\left[0,0,d_{3}-d_{2},0\right]$ and $D_{3}=\left[0,0,0,d_{4}-d_{1}\right]$, can be found to support the lemma, in which $D=D_{1}+D_{2}+D_{3}$, L=3, and K=4. □

**Figure 20:**
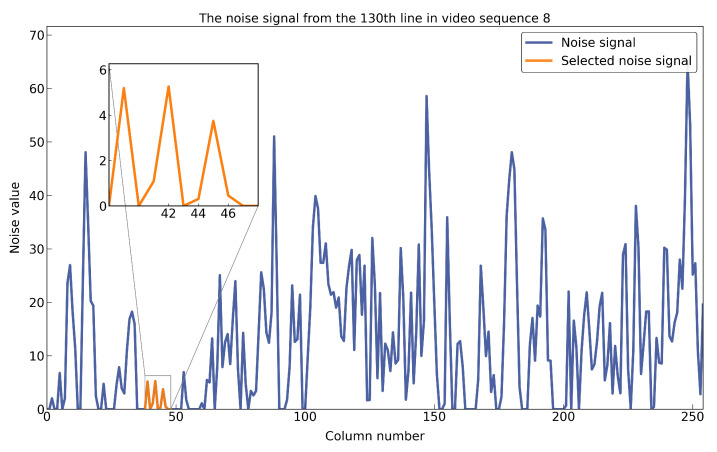
The blue line represents an example of a noisy signal, N, while the orange line shows the selected noise component, N’, in a magnified view.

Suppose noise is $N\in R^{m\times n\times 3}$, and a part of **N** is selected, namely the red signal $N^{\prime }\in R^{1\times 11}$, and $N^{\prime }=\left[0,5,0,1,5,0,0.3,3.9,0.4,0,0\right]$, as shown in Figure 20. Based on the lemma above, $N^{\prime }$ can be theoretically decomposed into the sum of several symmetrical signals, and symmetrical signals can be fitted with different Gaussian signals approximately. In this case, $N^{\prime }=N_{1}+N_{2}+N_{3}+N_{4}$, in which $N_{1}=\left[0,5,0,0,5,0,0,0,0,0,0\right]$, $N_{2}=\left[0,0,0,1,0,0,0,0,0,0,0\right]$, $N_{3}=\left[0,0,0,0,0,0,0.3,3.9,0.3,0,0\right]$, $N_{4}=\left[0,0,0,0,0,0,0,0,0.1,0,0\right]$. For example, $N_{1}$ can be fitted by Gaussian distribution $p\left(x\right)=5\exp\left(-\frac{{\left(x-1\right)}^{2}}{0.18}\right)$.

Suppose the noise in frame *i* is $X_{i}$, and its variance is $D(X_{i})$. In our experiment, we average 10 adjacent frames; thus, the denoisied data are $X=\frac{1}{10}\sum_{i=1}^{10}X_{i}$. By the property of the Gaussian distribution, namely $D\left(\alpha X\right)=\alpha^{2}D\left(X\right)$, the variance of *X* is $\frac{1}{100}\sum_{i=1}^{10}D\left(X_{i}\right)$, where α is a constant. When averaging 10 adjacent frames, the noise variance of each frame is reduced by a factor of 100. Assuming that the mean variance of noise in each channel is 300, 400, and 900, after averaging, the mean variance of noise changes to 3, 4, and 9, which greatly weakens the impact of noise.

### 5.3. Why Is Our Model Fast?

Our algorithm is very fast due to its complexity of O(kN), where *k* is a constant, and *N* is the block number.

Our algorithm consists of four parts. In the first part, the search exhausts all the steps, and the search radius is Sws. However, the search method is not a full search; thus, the search scope is ${\left(Sws+1\right)}^{2}$, and the total steps in the first part are ${\left(Sws+1\right)}^{2}N$.

The second part is vector updating. For each vector, Equation (3) consumes 16N steps, and Equations (4) and (5) consume 3N steps. Since the updating number is num, therefore, the maximum steps in the second part are num×19N.

The third part is a full search and the search area is ${\left(2SSws+1\right)}^{2}$, where SSws is the small search window size, so the total steps in the third part is ${\left(2SSws+1\right)}^{2}N$.

The fourth part is the denoising. In this part, the vectors are classified into three categories, and their numbers are $N_{1}$, $N_{2}$, and $N_{3}$, with $N_{1}+N_{2}+N_{3}=N$. For type 1 vectors, the system consumes $10N_{1}$ steps. For type 2 vectors, the system consumes $28N_{2}$ steps. For type 3 vectors, the system consumes $10N_{3}+{\left(2BSws+1\right)}^{2}N_{3}$ steps, where BSws is short for big search window size. The total steps in the fourth part are $10N_{1}+28N_{2}+10N_{3}+{\left(2BSws+1\right)}^{2}N_{3}$. For simplicity, the upper bound $(10+{\left(2BSws+1\right)}^{2})N$ is used as the total step count.

To sum up, the total steps are ${\left(Sws+1\right)}^{2}N+num\times 19N+{\left(2SSws+1\right)}^{2}N+\left(10+{\left(2BSws+1\right)}^{2}\right)N=kN$; thus, the complexity of our system is O(kN).

## 6. Ablation Study

### 6.1. Ablation on the Loop Count num as the Stopping Criterion

**Figure 21:**
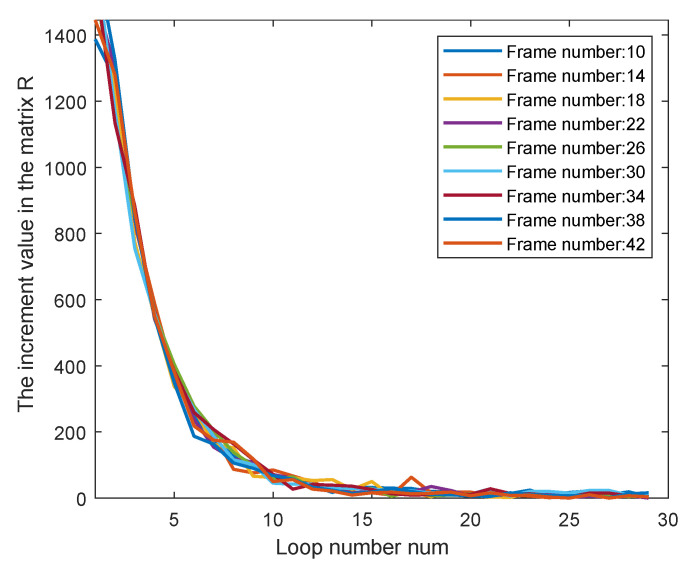
The ablation analysis of num to verify the appropriate stopping criterion.

In our method, num represents the number of loops, and a larger num results in more elements equaling 1 in the rationality matrix while also increasing the running time. Thus, num needs to be set appropriately to balance efficiency. The sum of the rationality matrix, namely $\sum_{a,b}R_{ab}$, is defined as its value. It can be observed that the increment in this matrix value gradually decreases with the increase of num. In Figure 21, different frames are sampled, and their increment curves nearly coincide. Due to the randomness of sampling, the distribution demonstrates strong statistical significance. It is clear that when num exceeds 15, it is difficult to bring obvious increment to the rationality matrix; thus, num is set as 15.

### 6.2. Ablation on β, the Proportion of Reasonable Vectors for Stopping

**Figure 22:**
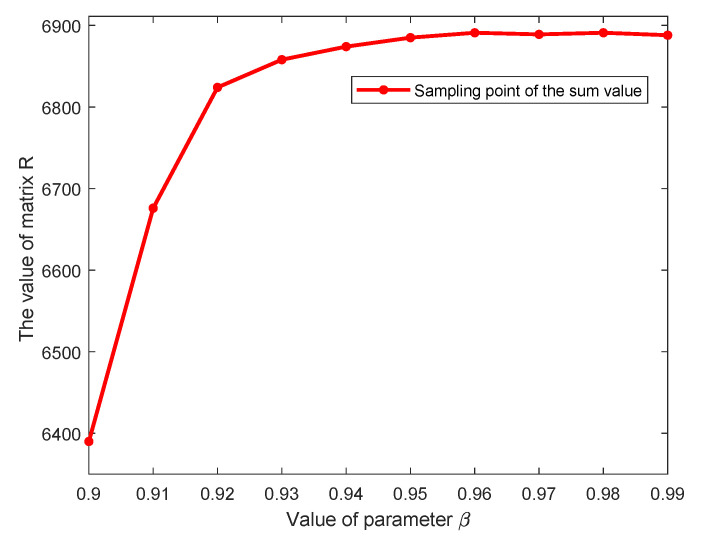
Ablation analysis of the relation between the value of the R matrix and the parameter β as the stopping criterion. The figure shows that after β exceeds 0.96, the increase in the R value is very limited.

In the experiment, block size Bs is set to 16. Therefore, if the all motion vectors are reasonable, the number of element 1 ($R_{ab}=1$) in the rationality matrix is $\frac{1920\times 1072\times 3}{16\times 16\times 3}=8040$. However, it is unrealistic for all motion vectors to be reasonable due to motion complexity. Some motion vectors converge to a stable value after many loop times (num>15). β is defined as $\frac{\sum_{a,b}R_{ab}}{8040}$, and Figure 22 shows the relationship between $\sum_{a,b}R_{ab}$ and β. Considering the convergence of $\sum_{a,b}R_{ab}$ and the time complexity of the system, parameter β is set as 0.96.

### 6.3. Ablation on Search Window Size for Search Scope

**Figure 23:**
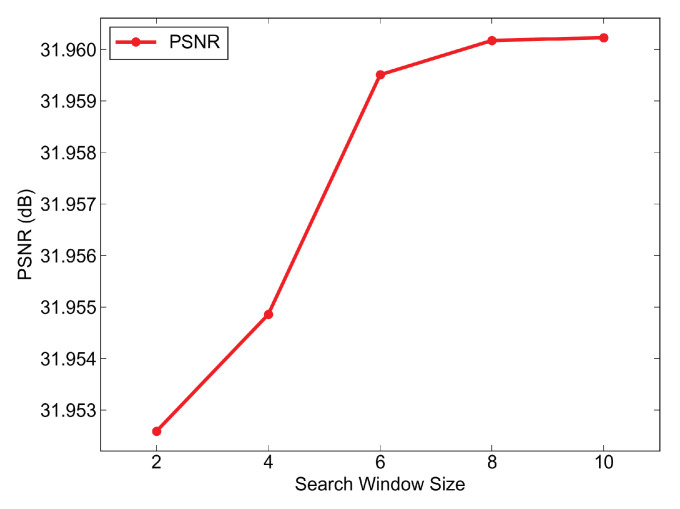
Analysis of search window size, as the PSNR improvement is very weak when the search window size exceeds 6.

The search window size controls the block matching range. Theoretically, a larger size increases the likelihood of matching similar blocks and improving performance. In our experiments, we varied sizes from 2 to 10 in steps of 2 and evaluated them using the PSNR. Figure 23 shows that the PSNR gains plateau beyond size 6. However, a larger range leads to increased computational and time costs. Thus, we selected 6 as the optimal balance point for the search window size.

### 6.4. Ablation on $\theta_{max}$ and $\theta_{min}$ Used as Phase Clamping Thresholds in Amplitude Phase Filter

**Figure 24:**
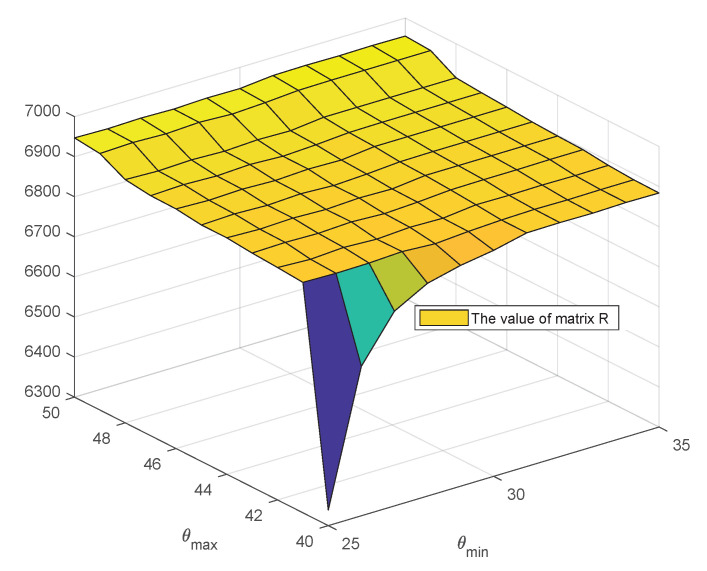
Ablation analysis of $\theta_{max}$ and $\theta_{min}$ for the appropriate division of reasonable vectors.

$\theta_{\max}$ and $\theta_{\min}$ are parameters defined in Equation (4). As visualized in Figure 24, the sum of the rationality matrix, denoted as $\sum_{a,b}R_{ab}$, increases with higher values of $\theta_{\max}$ and $\theta_{\min}$, and it eventually converges within a specific range ($44{}^{\circ}\leq \theta_{\max}\leq 48{}^{\circ}$, $29{}^{\circ}\leq \theta_{\min}\leq 35{}^{\circ}$). In this paper, the values of $\theta_{\max}$ and $\theta_{\min}$ are set to 45° and 30°, respectively. This configuration not only ensures the rationality of the matrix but also maintains stable system runtime performance.

### 6.5. Ablation on Comparing Motion Estimation Methods Used for Denoising

Our algorithm mainly consists of motion estimation and denoising. Consider two adjacent frames in the video, which are denoted as $f_{n}$ and $f_{n+1}$. The estimated motion vectors are used to artificially insert a new frame $f_{n+0.5}$ between $f_{n}$ and $f_{n+1}$. This frame $f_{n+0.5}$ is generated algorithmically to improve video smoothness, and its quality depends on the accuracy of motion estimation, thus serving as a means to evaluate motion estimation performance. For example, if the first frame $f_{1}$ and the third frame $f_{3}$ are used to interpolate the second frame ${\hat{f}}_{2}$, comparing ${\hat{f}}_{2}$ with the ground truth $f_{2}$ can measure the validity of the estimated motion vectors.

Several motion estimation algorithms are compared, such as BME (Bidirectional Motion Estimation) [47], FBJME (Forward–Backward Joint Motion Estimation) [56], DME (Dual Motion Estimation) [57], DSME (Direction-Select Motion Estimation) [58], and LQME (Linear Quadratic Motion Estimation) [59]. PSNR and SSIM are adopted as evaluation metrics to quantify the accuracy of motion vectors, and the dataset from the Experiments section is used. Table 4 shows that our method achieves first or second place in most cases, indirectly indicating that our motion estimation algorithm is effective and reasonable.

### 6.6. Ablation on How Component Changes Affect Speed

Our algorithm mainly includes four steps, namely coarse motion estimation, vector updating, vector refinement, and denoising. If step 1 is omitted, steps 2 and 3 cannot be performed. Similarly, step 4 is related to denoising, and omitting it would render the entire method ineffective. Therefore, the ablation study can only be conducted on steps 2 and 3 with four cases to consider.

Table 5 shows the results of the ablation study. In the experiment, only stationary sequences are used. Since no motion exists, omitting step 2 or step 3 has little impact on PSNR or SSIM values. For video sequences containing moving objects, only speed ablation experiments can be performed due to the lack of *ground truth*.

## 7. Limitations

Although our method achieves competitive results, it also has certain limitations. The first is that it is less effective at removing temporally correlated noise, such as certain types of Gaussian noise where the disturbance in each frame is nearly identical. Suppose the Gaussian noise in frame *i* is $X_{i}$, and $X_{i}\approx X_{j}$ for i≠j. When 10 adjacent frames are averaged, the noise variance $D\left(\frac{1}{10}\sum_{i=1}^{10}X_{i}\right)\approx D\left(\frac{1}{10}\times 10X_{i}\right)=D\left(X_{i}\right)$. Therefore, the averaging operation cannot suppress such noise. However, when the noise varies temporally, namely $X_{i}\neq X_{j}$ for i≠j, such as a in low-light environment, the averaging operation can suppress the noise. Second, it is difficult to capture some extremely complex or isolated motions, as the search radius in our method is finite. However, these motions are rare in the real world, justifying our decision to limit the search radius to a reasonable range.

## 8. Conclusions

Denoising low-light surveillance video presents a formidable challenge, stemming from the severe and complex noise patterns inherent in such conditions. The core difficulty lies in the critical trade-off between effectively suppressing intense noise and simultaneously preserving fine-grained texture details. Achieving this intricate balance is paramount for practical surveillance applications, as failure to do so can result in the loss of crucial visual information. In this paper, we proposed a tracking-based denoising algorithm designed for surveillance videos captured in extremely low-light environments. Our algorithm integrates coarse motion estimation via bilateral search for initial vector accuracy, motion vector updating using an amplitude-phase filter and rationality matrix to ensure local continuity, motion vector refinement with a small-area full search to achieve pixel-level precision without excessive computational cost, and a trilateral filter that combines spatial, pixel, and gradient domain information to effectively suppress noise by classifying motion vectors into three types and applying adaptive denoising strategies. Our method can effectively suppress noise while retaining detailed information, as demonstrated by extensive quantitative experiments and visual comparisons. In the future, we intend to explore other weighting dimensions for the trilateral filter, such as temporal consistency and texture complexity. Furthermore, we aim to replace the rigid three-category block classification with a more flexible, adaptive mechanism that allows for a smoother transition between categories.

## Figures and Tables

**Table 1:** Quantitative tests: average PSNR(dB)/SSIM values for the No. 1–7 video sequences, where **bold** and underlined texts indicate the best and second-best performance, respectively.

Methods’ Names	Video 1 PSNR/SSIM	Video 2 PSNR/SSIM	Video 3 PSNR/SSIM	Video 4 PSNR/SSIM	Video 5 PSNR/SSIM	Video 6 PSNR/SSIM	Video 7 PSNR/SSIM
**VBM4D** [15]	28.01/0.6650	27.04/ 0.5716	27.51/0.5931	28.97/ 0.6164	26.38/ 0.5874	28.87/0.6438	29.15/0.6197
**FastdvdNet** [24]	24.37/0.4629	23.30/0.2848	23.63/0.3474	24.46/0.3801	23.01/0.3221	24.54/0.3860	24.52/0.3778
**UDVD** [25]	26.15/0.5615	25.42/0.4109	25.93/0.4766	26.92/0.4871	25.11/0.4532	26.82/0.4895	27.14/0.4932
**FloRNN** [32]	24.82/0.4851	23.66/0.3044	24.04/0.3693	25.02/0.4049	23.44/0.3474	25.04/0.4119	25.09/0.4031
**RCD** [27]	28.22/0.6540	26.52/0.5160	27.44/0.5692	28.78/0.5892	26.11/0.5454	28.66/0.6065	28.90/0.5886
**ShiftNet** [28]	25.68/0.6548	26.73/ 0.5286	27.56/0.6079	28.69/ 0.6154	26.21/0.5744	27.34/0.6340	29.04/0.6188
**TAP** [39]	24.95/0.4969	23.99/0.3405	24.43/0.3916	24.80/0.4263	23.34/0.3775	25.54/0.4361	24.64/0.4287
**Turtle** [42]	23.30/0.4652	20.26/0.2581	21.99/0.3393	22.23/0.3640	20.87/0.3044	23.44/0.3902	22.01/0.3556
**VRT** [27]	25.84/0.5293	24.67/0.3614	25.08/0.4198	26.20/0.4518	24.29/0.3966	26.14/0.4612	26.29/0.4500
**Our method**	**31.14**/**0.7687**	**29.81**/**0.6524**	**30.28**/**0.7038**	**31.62**/**0.7131**	**29.03**/**0.6772**	**31.50**/**0.7220**	**31.75**/**0.7143**

**Table 2:** Quantitative tests: average PSNR(dB)/SSIM values for the No. 8–14 video sequences, where **bold** and underlined texts indicate the best and second-best performance, respectively.

Methods’ Names	Video 8 PSNR/SSIM	Video 9 PSNR/SSIM	Video 10 PSNR/SSIM	Video 11 PSNR/SSIM	Video 12 PSNR/SSIM	Video 13 PSNR/SSIM	Video 14 PSNR/SSIM
**VBM4D** [15]	29.79/0.7318	27.71/0.7250	24.01/0.7035	30.98/0.7483	28.94/0.7485	32.86/0.7899	28.02/0.7599
**FastdvdNet** [24]	28.57/0.7010	26.62/0.6927	23.37/0.6745	29.22/0.6840	26.85/0.6292	30.95/0.6985	25.93/0.5812
**UDVD** [26]	28.96/0.7169	27.16/0.7187	23.81/0.6990	29.70/0.7053	27.50/0.6703	30.85/0.6919	26.39/0.6136
**FloRNN** [32]	28.52/0.6968	26.63/0.6922	23.36/0.6740	29.21/0.6834	26.90/0.6327	30.85/0.6948	25.96/0.5830
**RCD** [27]	30.31/0.7704	28.01/0.7597	24.17/0.7351	31.70/0.7890	29.16/0.7807	33.85/0.8285	28.01/0.7809
**ShiftNet** [28]	29.25/0.6977	27.21/0.6752	23.62/0.6442	30.26/0.7044	28.91/0.7335	32.62/0.7927	28.21/0.7748
**TAP** [39]	28.28/0.6982	26.37/0.6919	23.35/0.6743	28.86/0.6975	26.66/0.6671	31.20/0.7293	26.12/0.6415
**Turtle** [42]	27.39/0.6644	25.76/0.6652	22.91/0.6494	27.92/0.6520	25.84/0.6037	29.54/0.6394	25.12/0.5420
**VRT** [27]	29.48/0.7463	27.27/0.7353	23.64/0.7092	30.40/0.7406	27.75/0.6917	32.21/0.7562	26.67/0.6475
**Our method**	**31.85**/**0.8467**	**29.50**/**0.8409**	**25.53**/**0.8112**	**33.02**/**0.8544**	**30.39**/**0.8306**	**34.69**/**0.8581**	**28.90**/**0.7925**

**Table 3:** Average speed (FPS) of different denoising algorithms.

Method	Speed (FPS)	Device	Method	Speed (FPS)	Device
VBM4D [15]	2.20	CPU	FastdvdNet [24]	129.87	GPU
UDVD [25]	20.58	GPU	FloRNN [32]	37.31	GPU
RCD [27]	67.56	GPU	ShiftNet [28]	0.33	GPU
TAP [39]	15.75	GPU	Turtle [42]	8.44	GPU
VRT [27]	1.05	GPU	Our method	1.17	CPU

**Table 4:** Quantitative comparison of different motion estimation algorithms on widely used video datasets. Results are reported as PSNR (dB) / SSIM. **bold** and underlined texts indicate the best and second-best performance, respectively.

Dataset	BME [46]	FBJME [55]	DME [56]	DSME [57]	LQME [58]	Our Method
Akiyo	44.26/0.9926	45.76/0.9950	47.20/0.9961	**47.39**/**0.9962**	46.61/0.9960	47.14/0.9956
Paris	34.24/0.9745	35.35/0.9795	36.42/0.9834	**36.80**/**0.9847**	36.14/0.9830	36.15/0.9822
Silent	34.54/0.9518	35.35/**0.9795**	35.88/0.9636	36.11/0.9656	−/−	**36.13**/0.9645
Crew	28.46/0.8328	31.15/0.8962	31.05/0.8938	31.59/0.9067	−/−	**31.91**/**0.9074**
Foreman	28.65/0.8636	31.72/0.8991	32.64/0.8939	33.15/0.9042	32.64/0.9049	**33.70**/**0.9334**
Football	22.58/0.6981	23.16/0.7125	22.49/0.6803	22.86/0.7046	22.96/0.6689	**23.90**/**0.7549**
Mobile	20.63/0.7095	27.61/0.9383	28.31/0.9435	28.63/**0.9602**	27.71/0.9438	**29.68**/0.9558
Soccer	23.48/0.7552	25.30/0.8154	24.32/0.7808	24.89/0.8071	−/−	**26.70**/**0.8487**
**Average**	29.61/0.8473	31.93/0.9019	32.28/0.8919	32.68/0.9036	−/−	**33.16**/**0.9178**

**Table 5:** Impact of different step combinations.

Case	Step 1	Step 2	Step 3	Step 4	Speed/s
Case 1	✓	×	×	✓	0.903
Case 2	✓	×	✓	✓	1.123
Case 3	✓	✓	×	✓	1.026
Case 4	✓	✓	✓	✓	1.169

## Data Availability

The data presented in this study are available on request from the corresponding author.

## References

[1] Malyugina A., Anantrasirichai N., Bull D. (2025). Wavelet-based topological loss for low-light image denoising. Sensors.

[2] Liu X., Zhao Q. (2025). Guided filter-inspired network for low-light RAW image enhancement. Sensors.

[3] Lee S.-Y., Rhee C.E. (2019). Motion estimation-assisted denoising for an efficient combination with an HEVC encoder. Sensors.

[4] Fu Y., Wang Z., Zhang T., Zhang J. (2023). Low-light raw video denoising with a high-quality realistic motion dataset. IEEE Trans. Multimedia.

[5] Im Y., Pak J., Na S., Park J., Ryu J., Moon S., Koo B., Kang S.-J. (2025). Supervised denoising for extreme low-light raw videos. IEEE Trans. Circuits Syst. Video Technol..

[6] Yamamoto H., Anami S., Matsuoka R. (2024). Optimizing dynamic mode decomposition for video denoising via plug-and-play alternating direction method of multipliers. Signals.

[7] Dabov K., Foi A., Katkovnik V., Egiazarian K. Image Denoising with Block-Matching and 3D Filtering. Proceedings of the SPIE Electronic Imaging 2006: Image Processing.

[8] He K., Sun J., Tang X. (2013). Guided image filtering. IEEE Trans. Pattern Anal. Mach. Intell..

[9] Chan T.W., Au O.C., Chong T.S., Chau W.S. A Novel Content-Adaptive Video Denoising Filter. Proceedings of the 2005 IEEE International Conference on Acoustics, Speech, and Signal Processing.

[10] Selesnick I.W., Li K.Y. Video denoising using 2D and 3D dual-tree complex wavelet transforms. Proceedings of the Wavelets: Applications in Signal and Image Processing X.

[11] Jovanov L., Pizurica A., Schulte S., Schelkens P., Munteanu A., Kerre E., Philips W. (2009). Combined Wavelet-Domain and Motion-Compensated Video Denoising Based on Video Codec Motion Estimation Methods. IEEE Trans. Circuits Syst. Video Technol..

[12] Dugad R., Ahuja N. Video Denoising by Combining Kalman and Wiener Estimates. Proceedings of the 1999 International Conference on Image Processing.

[13] Buades A., Lisani J.-L., Miladinovic M. (2016). Patch-Based Video Denoising with Optical Flow Estimation. IEEE Trans. Image Process..

[14] Dabov K., Foi A., Katkovnik V., Egiazarian K. Image Restoration by Sparse 3D Transform-Domain Collaborative Filtering. Proceedings of the SPIE Electronic Imaging 2008: Image Processing: Algorithms and Systems VI.

[15] Maggioni M., Boracchi G., Foi A., Egiazarian K. (2012). Video Denoising, Deblocking, and Enhancement Through Separable 4-D Nonlocal Spatiotemporal Transforms. IEEE Trans. Image Process..

[16] Arias P., Morel J.-M. (2017). Video Denoising via Empirical Bayesian Estimation of Space-Time Patches. J. Math. Imaging Vis..

[17] Vaksman G., Elad M., Milanfar P. Patch Craft: Video Denoising by Deep Modeling and Patch Matching. Proceedings of the 2021 IEEE/CVF International Conference on Computer Vision.

[18] Davy A., Ehret T., Morel J.-M., Arias P., Facciolo G. A Non-Local CNN for Video Denoising. Proceedings of the 2019 IEEE International Conference on Image Processing.

[19] Davy A., Ehret T., Morel J.-M., Arias P., Facciolo G. (2020). Video Denoising by Combining Patch Search and CNNs. J. Math. Imaging Vis..

[20] Qu Y., Zhou J., Qiu S., Xu W., Li Q. Recursive Video Denoising Algorithm for Low Light Surveillance Applications. Proceedings of the 2021 14th International Congress on Image and Signal Processing, BioMedical Engineering and Informatics.

[21] Kim M., Park D., Han D., Ko H. (2015). A Novel Approach for Denoising and Enhancement of Extremely Low-Light Video. IEEE Trans. Consum. Electron..

[22] Krizhevsky A., Sutskever I., Hinton G.E. (2017). ImageNet classification with deep convolutional neural networks. Commun. ACM.

[23] Tassano M., Delon J., Veit T. DVDNET: A Fast Network for Deep Video Denoising. Proceedings of the 2019 IEEE International Conference on Image Processing.

[24] Tassano M., Delon J., Veit T. FastDVDnet: Towards real-time deep video denoising without flow estimation. Proceedings of the 2020 IEEE/CVF Conference on Computer Vision and Pattern Recognition.

[25] Sheth D.Y., Mohan S., Vincent J.L., Manzorro R., Crozier P.A., Khapra M.M., Simoncelli E.P., Fernandez-Granda C. Unsupervised Deep Video Denoising. Proceedings of the 2021 IEEE/CVF International Conference on Computer Vision.

[26] Qi C., Chen J., Yang X., Chen Q. Real-time Streaming Video Denoising with Bidirectional Buffers. Proceedings of the 30th ACM International Conference on Multimedia.

[27] Zhang Z., Jiang Y., Shao W., Wang X., Luo P., Lin K., Gu J. Real-Time Controllable Denoising for Image and Video. Proceedings of the 2023 IEEE/CVF Conference on Computer Vision and Pattern Recognition.

[28] Li D., Shi X., Zhang Y., Cheung K.C., See S., Wang X., Qin H., Li H. A Simple Baseline for Video Restoration with Grouped Spatial-Temporal Shift. Proceedings of the 2023 IEEE/CVF Conference on Computer Vision and Pattern Recognition.

[29] Shi X., Huang Z., Bian W., Li D., Zhang M., Cheung K.C., See S., Qin H., Dai J., Li H. VideoFlow: Exploiting Temporal Cues for Multi-frame Optical Flow Estimation. Proceedings of the 2023 IEEE/CVF International Conference on Computer Vision.

[30] Chen Z., Jiang T., Hu X., Zhang W., Li H., Wang H. Spatiotemporal Blind-Spot Network with Calibrated Flow Alignment for Self-Supervised Video Denoising. Proceedings of the Thirty-Ninth AAAI Conference on Artificial Intelligence.

[31] Wang X., Chan K.C.K., Yu K., Dong C., Loy C.C. EDVR: Video Restoration With Enhanced Deformable Convolutional Networks. Proceedings of the 2019 IEEE/CVF Conference on Computer Vision and Pattern Recognition Workshops.

[32] Li J., Wu X., Niu Z., Zuo W. Unidirectional Video Denoising by Mimicking Backward Recurrent Modules with Look-Ahead Forward Ones. Proceedings of the European Conference on Computer Vision.

[33] Chen X., Song L., Yang X. Deep RNNs for Video Denoising. Proceedings of the Applications of Digital Image Processing XXXIX.

[34] Wang Y., Bai X. (2023). Versatile recurrent neural network for wide types of video restoration. Pattern Recognit..

[35] Maggioni M., Huang Y., Li C., Xiao S., Fu Z., Song F. Efficient Multi-Stage Video Denoising with Recurrent Spatio-Temporal Fusion. Proceedings of the 2021 IEEE/CVF Conference on Computer Vision and Pattern Recognition.

[36] Liang J., Fan Y., Xiang X., Ranjan R., Ilg E., Green S., Cao J., Zhang K., Timofte R., Van Gool L. Recurrent video restoration transformer with guided deformable attention. Proceedings of the 36th Conference on Neural Information Processing Systems.

[37] Yue H., Cao C., Liao L., Yang J. (2025). RViDeformer: Efficient Raw Video Denoising Transformer with a Larger Benchmark Dataset. IEEE Trans. Circuits Syst. Video Technol..

[38] Aiyetigbo M., Ravichandran D., Chalhoub R., Kalivas P., Luo F., Li N. Unsupervised Coordinate-Based Video Denoising. Proceedings of the 2024 IEEE International Conference on Image Processing.

[39] Fu Z., Guo L., Wang C., Wang Y., Li Z., Wen B. Temporal As a Plugin: Unsupervised Video Denoising with Pre-trained Image Denoisers. Proceedings of the European Conference on Computer Vision. Cham: Springer Nature Switzerland.

[40] Zheng H., Pang T., Ji H. Unsupervised Deep Video Denoising with Untrained Network. Proceedings of the Thirty-Seventh AAAI Conference on Artificial Intelligence.

[41] Laine S., Karras T., Lehtinen J., Aila T. High-Quality Self-Supervised Deep Image Denoising. Proceedings of the 33rd Conference on Neural Information Processing Systems.

[42] Ghasemabadi A., Janjua M.K., Salameh M., Niu D. Learning Truncated Causal History Model for Video Restoration. Proceedings of the 38th Conference on Neural Information Processing Systems.

[43] Jin Y., Ma X., Zhang R., Chen H., Gu Y., Ling P., Chen E. (2025). Masked Video Pretraining Advances Real-World Video Denoising. IEEE Trans. Multimed..

[44] Dewil V., Anger J., Davy A., Ehret T., Facciolo G., Arias P. Self-supervised Training for Blind Multi-Frame Video Denoising. Proceedings of the 2021 IEEE Winter Conference on Applications of Computer Vision.

[45] Lee S., Cho D., Kim J., Kim T.H. Restore from Restored: Video Restoration with Pseudo Clean Video. Proceedings of the 2021 IEEE/CVF Conference on Computer Vision and Pattern Recognition.

[46] Xu P., Zheng P., Zheng L., Zhang X., Shang Y., Zhang H., Geng Y., Gao J., Jiang H., Dong J., Zhang L., Cheng D. (2024). Denoising Real-World Low Light Surveillance Videos Based on Trilateral Filter. Proceedings of the 2nd International Conference on Internet of Things, Communication and Intelligent Technology.

[47] Choi B.T., Lee S.H., Ko S.J. (2000). New Frame Rate Up-Conversion Using Bi-Directional Motion Estimation. IEEE Trans. Consumer Electron..

[48] Guo D., Lu Z. (2016). Motion-Compensated Frame Interpolation with Weighted Motion Estimation and Hierarchical Vector Refinement. Neurocomputing.

[49] Tomasi C., Manduchi R. Bilateral Filtering for Gray and Color Images. Proceedings of the Sixth International Conference on Computer Vision.

[50] Paris S., Durand F. (2007). A Fast Approximation of the Bilateral Filter Using a Signal Processing Approach. Int. J. Comput. Vis..

[51] Chen D., Ardabilian M., Chen L. Depth Edge Based Trilateral Filter Method for Stereo Matching. Proceedings of the 2015 IEEE International Conference on Image Processing (ICIP).

[52] Stoll M., Volz S., Bruhn A. Joint Trilateral Filtering for Multiframe Optical Flow. Proceedings of the 2013 IEEE International Conference on Image Processing.

[53] Pont-Tuset J., Perazzi F., Caelles S., Arbeláez P., Sorkine-Hornung A., Van Gool L. (2017). The 2017 DAVIS Challenge on Video Object Segmentation. arXiv.

[54] Yue H., Cao C., Liao L., Chu R., Yang J. Supervised Raw Video Denoising With a Benchmark Dataset on Dynamic Scenes. Proceedings of the 2020 IEEE/CVF Conference on Computer Vision and Pattern Recognition.

[55] Fu H., Zheng W., Wang X., Wang J., Zhang H., Ma H. Dancing in the Dark: A Benchmark towards General Low-light Video Enhancement. Proceedings of the 2023 IEEE/CVF International Conference on Computer Vision.

[56] Vinh T.Q., Kim Y.-C., Hong S.-H. Frame Rate Up-Conversion Using Forward-Backward Jointing Motion Estimation and Spatio-Temporal Motion Vector Smoothing. Proceedings of the 2009 International Conference on Computer Engineering & Systems.

[57] Kang S.J., Yoo S., Kim Y.H. (2010). Dual Motion Estimation for Frame Rate Up-Conversion. IEEE Trans. Circuits Syst. Video Technol..

[58] Yoo D.G., Kang S.J., Kim Y.H. (2013). Direction-Select Motion Estimation for Motion-Compensated Frame Rate Up-Conversion. J. Display Technol..

[59] Guo Y., Chen L., Gao Z., Zhang X. (2015). Frame Rate Up-Conversion Using Linear Quadratic Motion Estimation and Trilateral Filtering Motion Smoothing. J. Display Technol..

